# Energy Storage Behavior of Lithium-Ion Conducting poly(vinyl alcohol) (PVA): Chitosan(CS)-Based Polymer Blend Electrolyte Membranes: Preparation, Equivalent Circuit Modeling, Ion Transport Parameters, and Dielectric Properties

**DOI:** 10.3390/membranes10120381

**Published:** 2020-11-30

**Authors:** Mohamad Brza, Shujahadeen B. Aziz, Salah Raza Saeed, Muhamad H. Hamsan, Siti Rohana Majid, Rebar T. Abdulwahid, Mohd F. Z. Kadir, Ranjdar M. Abdullah

**Affiliations:** 1 Manufacturing and Materials Engineering Department, Faculty of Engineering, International Islamic University of Malaysia, Kuala Lumpur 50603, Malaysia; mohamad.brza@gmail.com; 2 Hameed Majid Advanced Polymeric Materials Research Laboratory, Physics Department, College of Science, University of Sulaimani, Qlyasan Street, Sulaimani 46001, Iraq; rebar.abdulwahid@univsul.edu.iq (R.T.A.); ranjdar.abdullah@univsul.edu.iq (R.M.A.); 3 Department of Civil Engineering, College of Engineering, Komar University of Science and Technology, Sulaimani 46001, Iraq; 4 Charmo Research Center, Charmo University, Peshawa Street, Chamchamal 46023, Iraq; salah.saeed@charmouniversity.org; 5 Centre for Foundation Studies in Science, University of Malaya, Kuala Lumpur 50603, Malaysia; hafizhamsan93@gmail.com (M.H.H.); mfzkadir@um.edu.my (M.F.Z.K.); 6 Centre for Ionics University of Malaya, Department of Physics, Faculty of Science, University of Malaya, Kuala Lumpur 50603, Malaysia; shana@um.edu.my; 7 Department of Physics, College of Education, Old Campus, University of Sulaimani, Kurdistan Regional Government, Sulaimani 46001, Iraq

**Keywords:** PVA:CS polymer blends, electrical equivalent circuit, impedance study, ion transport properties, dielectric properties, energy storage study

## Abstract

Plasticized lithium-ion-based-conducting polymer blend electrolytes based on poly(vinyl alcohol) (PVA):chitosan (CS) polymer was prepared using a solution cast technique. The conductivity of the polymer electrolyte system was found to be 8.457 × 10^−4^ S/cm, a critical factor for electrochemical device applications. It is indicated that the number density (*n*), diffusion coefficient (*D*), and mobility (*μ*) of ions are increased with the concentration of glycerol. High values of dielectric constant and dielectric loss were observed at low frequency region. A correlation was found between the dielectric constant and DC conductivity. The achieved transference number of ions (t_ion_) and electrons (t_e_) for the highest conducting plasticized sample were determined to be 0.989 and 0.011, respectively. The electrochemical stability for the highest conducting sample was 1.94 V, indicated by linear sweep voltammetry (LSV). The cyclic voltammetry (CV) response displayed no redox reaction peaks through its entire potential range. Through the constructing electric double-layer capacitor, the energy storage capacity of the highest conducting sample was investigated. All decisive parameters of the EDLC were determined. At the first cycle, the specific capacitance, internal resistance, energy density, and power density were found to be 130 F/g, 80 Ω, 14.5 Wh/kg, and 1100 W/kg, respectively.

## 1. Introduction

The use of environmentally friendly electrolytes is one of the hot topics in secondary lithium batteries studies. Solid polymer electrolytes (SPEs) have been under intensive study despite being familiarized with these electrolytes 40 years ago [1]. Verma et al. [2] fabricated nanocomposite polymer electrolyte of 95(70PEO:30AgI):5SiO_2_ with the high conductivity of 2.5 × 10^−3^ S/cm. The authors indicated that the polymer electrolyte is suitable for solid state capacitor applications due to the conductivity enhancement and improvement in amorphous structure.

SPEs are composed of polymer hosts enriched with polar groups where salt is dissolved, showing desirable properties [3]—such as light weight, high flexibility factor, and lack of leakage [4,5]. It is also worth mentioning that these polymer electrolytes are superior over inorganic solids in terms of flexibility [6,7]. The inorganic solid electrolyte is appropriate for rigid battery design, as it has higher mechanical strength, better chemical/thermal stabilities, and shows a clear conductivity advantage over a wide range of temperatures. Nevertheless, the sulfide-based inorganic electrolyte chemical stability has to be more developed; whereas, for NASICON (NA super ion conductor)-based inorganic electrolyte, its wetting with sodium metal must be improved, so as to enhance the cycling life and energy density in the resultant sodium-ion battery with metallic sodium as anode [8]. Natural SPEs—mainly starch, cellulose, chitosan (CS), carrageenan, and agarose—have recently drawn significant attention. This is because of their potential uses in electrochemical devices, such as electrochromic devices, high energy density batteries, sensors, EDLC, and fuel cells [9,10,11]. These natural polymers are featured by biodegradability, environmental friendliness, and easy handling during preparation [12,13]. CS is the second abundant natural biopolymer after cellulose [14,15]. It is obtained from the extraction process where shrimp waste has been used as a source enriched in CS [16,17]. CS is a polycationic polymer that within each monomer unit contains one amino and two hydroxyl groups [18]. Natural polymers have been focused on their high biocompatibility, biodegradability, low toxicity, and cost-effectiveness [19,20]. A qualified candidate to be used as a host polymer in the electrolyte preparation is the semi-crystalline poly (vinyl alcohol) (PVA). Nowadays, PVA has been used in several energy devices, including direct methanol fuel cells, Zn–air batteries and rechargeable Ni–MH batteries [21,22,23]. PVA provides exceptional properties, for instance, high dielectric strength, plausible charge-storage capacity, and film-forming capacity. It is also a hydrophilic material that has a high density of reactive functional groups [24,25]. The functional groups are essential for the blending of a polymer with CS polymer. This methodology has attracted the attention of many researcher groups in which new materials can be prepared. The blended polymers exhibit superior properties compared to the individual polymers [26,27]. The blended polymers are copolymers that contain two polymers which combined via secondary forces, such as van der Waals and H-bonding [28,29]. An earlier study has shown that improving the conductivity of a particular polymer electrolyte can be achieved via blending with another polymer [27]. In dealing with the conductivity of polymer electrolytes, the ion relaxation and charge transport mechanism have to be evaluated [30]. According to fundamental principles, there has been no complete understanding of the mechanism of ionic conduction within the polymer. This is due to the complexity of the process of ionic conduction within these materials in which several factors contribute—for example, degree of salt dissociation, salt concentration, the dielectric constant of host polymer, the tendency of ion to aggregate, and polymer chains’ mobility [31,32,33,34]. The analysis of ionic conductivity of polymer electrolytes via dielectric properties provides insight into the eligibility of polymer of interest in large scale. It is also well-known that the study of dielectric relaxation phenomena leads to understanding ion transport mechanisms, including the extent of ionic/molecular interaction in SPE systems [35].

In this work, lithium perchlorate (LiClO_4_) was used as a salt owing to its low lattice energy. LiClO_4_ consists of a large size anion a small size cation. The ionic conductivity is influenced by the diffusion rate of cations and anions which depends on the ion size. LiClO_4_ is selected owing to its good conductivity and ease of complex creation in the fabrication of SPEs [36]. Therefore, LiClO_4_ is more promising in comparison with other lithium salts owing to the low interfacial resistance when lithium metal was used as anode. Moreover, LiClO_4_ possesses large dissociation energy, hence it is a good soluble in most solvents [37]. In this work, glycerol is also used as plasticizer to enhance ionic conductivity. Pawlicka et al. [38] documented an increase from ∼10^−8^ to 10^−4^ S cm^−1^ with the help of glycerol in their electrolyte systems. High dielectric constant of glycerol weakens the electrostatic force among the cations and anions of the salt which can create more mobile ions. The plasticizer can also enhance the amorphous structure of the electrolyte [39].

Electrochemical double-layer capacitor (EDLC) is a device where the energy storage mechanism occurs owing to accumulation of ions at the interfaces between the blocking electrodes and electrolytes. EDLCs have long lifetimes, high power density, fast charge/discharge and identical carbonaceous electrodes [40,41,42]. There are many reports that used polymer electrolytes in EDLC fabrication [40,41,42]. In this study, the highest conducting electrolyte was employed in the fabrication of EDLCs. In the current work, the dielectric properties and relaxation dynamics of polymer electrolytes based on PVA:CS:LiClO_4_ plasticized with glycerol have been analyzed for the first time. Here, the dielectric and electrical modulus properties of plasticized polymer electrolyte PVA:CS:LiClO_4_ and its energy storage capacity (i.e., electric double-layer capacitor) have been studied.

## 2. Experimental

### 2.1. Materials and Preparation of Blend SPE Films

The chitosan (CS) with average molecular weight of 310,000–375,000 g mol^−1^, PVA with an average molecular weight of 35000 g mol^−1^, Lithium perchlorate (LiClO_4_) salt, and acetic acid (CH_3_COOH) solution have been used in the preparation of PVA:CS: LiClO_4_ blend SPE films. The solution cast technique was used in the film preparation.

All chemicals that were used as obtained were purchased from the (Sigma-Aldrich, Kuala Lumpur, Malaysia). In this work, 0.5 g of CS was dissolved in 30 mL of the solution of 1% acetic acid (CH_3_COOH) and then stirred at room temperature for 3 h. 0.5 g of PVA was dissolved in 20 mL of distilled water at 80 °C during PVA preparation. The solution was left at room temperature to cool down. Then, both solutions were mixed with continuous stirring using a magnetic stirrer. Afterwards, 40 wt % of lithium perchlorate (LiClO_4_) salt as a dopant was added into the solution mixture with stirring continuously until a clear solution was gained. To plasticization of the solution mixture, various quantities of glycerol were added ranged from 14 to 42 wt %. Based on the addition amount of 14 wt %, 28 wt %, and 42 wt % of glycerol to the PVA:CS:LiClO_4_ system, the prepared polymer blend electrolyte samples were coded as PVCSG1, PVCSG2, PVCSG3, respectively. Mixtures of the final solution were eventually poured into dry and clean glass Petri dishes. They were left to evaporate gradually at room temperature. Thereby, dry and free-standing of PVA:CS:LiClO_4_:glycerol blend SPE films were obtained. The highest conducting plasticized electrolyte system (PVCSG3) was used for electric double-layer capacitor (EDLC) device application.

### 2.2. Impedance and Equivalent Circuit Modeling Studies (EIS)

In the analysis of electrochemical properties of materials utilized in solid-state batteries, complex impedance spectroscopy (CIS) can be used as a powerful technique [32,33,34]. This approach provides insight into the electrical properties of bulk materials as well as the interface region of the electronically conductive electrodes. The small discs as electrode separator of 2 cm in diameter from SPE films were constructed and then sandwiched between two stainless steel electrodes using spring pressure. Impedance measurements of the films were carried out using the HIOKI 3531 Z Hi-tester (Nagano, Japan) in a frequency range of 50–5000 kHz. The measurements were managed by customized software, in which the impedance spectrum with real and imaginary parts could be given. The impedance spectra were exhibited in the Nyquist plot. The bulk resistance (*R_b_*) values were obtained from the intercept of the spike with the real part on the x-axis of the spectra. The conductivity values were calculated from the relationship shown below [32]

(1) $$\sigma_{dc}\;=\;\left(\frac{1}{R_{b}}\right)\times \left(\frac{t}{A}\right)$$

where; *t* and *A* are the thickness and area of the film, respectively.

The real (*Z_r_*) and imaginary (*Z_i_*) parts of the complex impedance (Z*) were used for the evaluation of dielectric constant using the equations [29]

(2) $$\varepsilon^{\prime }=\frac{Z_{i}}{\omega \;C_{o}(Z_{r}{}^{2}+Z_{i}{}^{2})}$$

(3) $$\varepsilon^{\prime \prime }=\frac{Z^{\prime }}{\omega C_{o}\left({Z^{\prime }}^{2}+{Z^{\prime \prime }}^{2}\right)}$$

where *C_o_* is the vacuum capacitance determined from *ε_o_A/t.* The *ε_o_* is the permittivity of free space (8.85 × 10^−12^ F/m). The angular frequency ω is equivalent to *ω = 2πf*, where; *f* is the applied field frequency. The Z_r_ and Z_i_ data were collected from the EIS data and then used to determine the ɛ’ and ɛ” data.

### 2.3. TNM and LSV Analyses

To obtain the ionic and electronic transference numbers (t_ion_ and t_e_), the V & A instrument DP3003 digital control DC power supply was used by DC polarization method. The highest conducting plasticized sample (i.e., PVCSG3) was placed between a pair of stainless steel (SS) blocking electrodes. A 0.2 V constant working voltage was applied to polarize the cell at room temperature. To see the highest working voltage of the PVCSG3 system, the linear sweep voltammetry (LSV) was done using a Digi-IVY DY2300 potentiostat. In the voltage range of 0 to 2.5V, the LSV was measured at a scan rate of 10 mV/s.

### 2.4. Construction and Characterization of EDLC

To construct the EDLC for testing, the carbon electrodes were prepared first. For the blending of 6.25% carbon black powder and 81.25% activated carbon, a planetary ball miller was used. Meanwhile, in 15 mL of N-methyl pyrrolidone (NMP), 12.5 wt % of polyvinylidene fluoride (PVdF) was dissolved. Subsequently, the mixture of the powders was poured into the PVdF-NMP solution followed by stirring before a thick black solution emerged. Then, the black solution was coated with a doctor’s blade on an aluminum foil (AF). The drying process for the coated AF was carried out at a temperature of 60 °C in an oven. Ultimately, the electrodes were stored in a desiccator to make sure of dryness before measurements. In order to calculate the decisive parameters of the EDLC, such as equivalent series resistance (*R_es_*), specific capacitance from charge–discharge (*C_CD_*), energy (*E*), and power density (*P*), the current density was kept at 0.5 mA/cm^2^.

## 3. Results and Discussion

### 3.1. Impedance and Electrical Equivalent Circuit Studies

**Figure 1:**
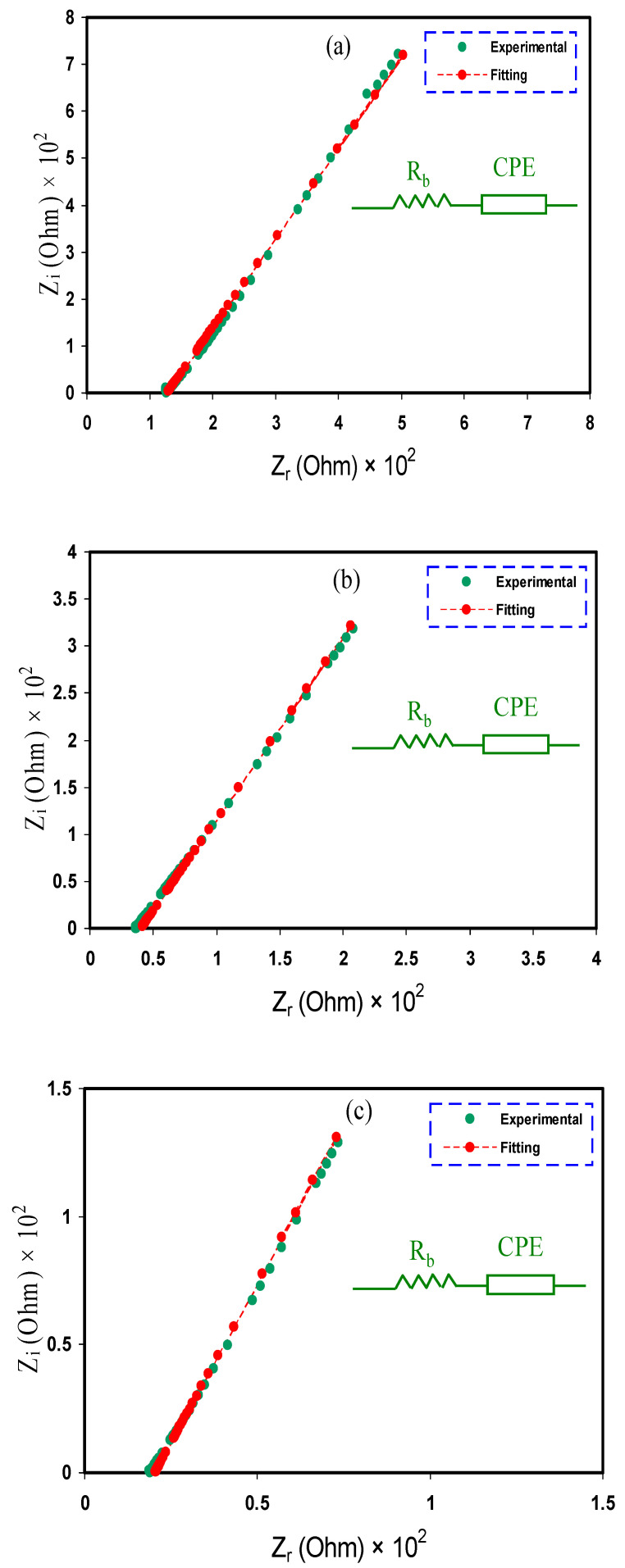
EIS plots for (**a**) PVCSG1, (**b**) PVCSG2, and (**c**) PVCSG3 electrolyte films.

It is well known that two factors, the number of ion carriers and their mobility determine the materials and polymers’ ionic conductivity. To deal with these factors in polymer-based electrolytes, impedance spectroscopy is a candidate that characterized by simplicity and powerfulness [43,44]. During data analysis, a suitable electrical equivalent circuit (EEC) model was suggested. The EEC as quick and straightforward is often used in the analysis of impedance data points that provides a comprehensive picture of the whole system [45,46]. The resistance and circuit components of the EEC modeling are associated with the samples’ electrical properties. The fitting process of the impedance spectra with the EEC model for all the plasticized samples is presented in Figure 1a–c. It is seen that the impedance of the films is a spike-like shape.

The EDLC at the electrode/electrolyte interface in an electrochemical cell is created by the motion of cations and anions toward the electrodes in opposite directions. The blocking of the electrode surface was taken into consideration in the impedance analysis. In the impedance spectra, the indication of capacitor system should appear in the form of a vertical spike (by presuming it is ideal) [32,47]. The creation of double-layer capacitance between the electrodes and the electrolyte blend in the form of constant phase element (CPE) can be recognized from the low-frequency region response. In the equivalent circuit of electrochemical processes, CPE is usually used other than the ideal capacitor in the real system [48]. The plasticized electrolytes’ spike feature indicates that the polymer modeling’s resistive component is predominated [9,49].

The Z_CPE_ impedance can be written as [31,48]:

(4) $$Z_{CPE}=\frac{1}{C\omega^{n}}\left[\cos\left(\frac{\pi n}{2}\right)-i\sin\left(\frac{\pi n}{2}\right)\right]$$

In this case, the values of *Z_r_* and *Z_i_* are correlated with the EEC, which can be expressed, mathematically, as

(5) $$Z_{r}=R+\frac{\cos(\pi n/2)}{C\omega^{n}}$$

(6) $$Z_{i}=\frac{\sin(\pi n/2)}{C\omega^{n}}$$

where *C* is the CPE capacitance, ω is the angular frequency, and *n* is related to the deviation in the complex impedance spectra at the vertical axis. The EEC fitting parameters are shown in Table 1.

Table 2 presents the DC conductivity values at room temperature for the plasticized polymer blend electrolytes. It is seen that the value of DC conductivity increases with increasing the quantity of glycerol quantity. The relatively high DC conductivity value of about 8.457 × 10^−4^ S/cm is seen for the sample incorporated with 42 wt % glycerol. Solvent-free polymer electrolytes with DC ionic conductivities (σ_dc_) have been previously documented in the 10^−5^–10^−2^ S/cm range at room and different temperatures [50,51,52,53]. Cevik and Bozkurt [42] synthesized polymer metal electrolyte poly(acrylic acid) (PAA)-cobalt sulfate (Co) with the DC ionic conductivity of 3.15 × 10^−4^ S/cm. The authors used the electrolyte for application in EDLC with high performance. It is possible to rationalize the contribution of both the segmental motion and ionic conductivity to the whole conductivity in various ways. Herein, a mathematical relationship between DC conductivity(*σ_dc_*) on one side and charge (*q_i_*), charge carrier concentration (*n_i_*), and the charge carrier mobility (*µ_i_*) on other can be expressed from the well-known equation [50],

σ = *Σ n_i_q_i_ µ_i_* (7)

From the above equation, it is obvious that the increase in DC conductivity is mainly governed by the number of ions rather than ion mobility at room temperature. On the one hand, the number of ions is controlled by the salt concentration; on the other hand, mobility has been increased through glycerol as a plasticizer.

As the impedance data consists of a spike only, the ionic transport parameters of mobility (*μ*), diffusion coefficient (*D*), and number density (*n*) of ions were obtained using the following equations, which are obtained in detail in [48,54,55,56].

The *D* of the ion carriers of the systems can be obtained by the equation below,

(8) $$D=D_{o}\;exp\{-0.0297{\left[ln\;D_{o}\right]}^{2}-1.4348\;ln\;D_{o}-14.504\}$$

where

(9) $$D_{o}\;=\left(\frac{4k^{2}l^{2}}{R_{b}{}^{4}\omega 3_{min}}\right)$$

where l is the electrolyte thickness and *ω_min_* is the angular frequency, which corresponds to the minimum *Z_i_*.

The mobility (*µ*) of the ion carriers can be obtained from the following equation,

(10) $$\mu \;=\left(\frac{eD}{K_{b}T}\right)$$

where *T* is the absolute temperature and *k_b_* is the Boltzmann constant.

For better understanding, the diffusion coefficient and mobility for each cation and anion were determined and listed them in Table 3 and also explained in detail in the next sections.

Since DC conductivity of ions is shown by

(11) $$\sigma_{Dc}=ne\mu$$

Thus, the number density of ion carriers (*n*) is obtained by Equation (11).

Table 3 indicates the ion transport parameters and the ω_min_ values for the systems. Based on Table 3, it is observed that the D value increases as the amount of glycerol increase from 14 to 42 wt %. The same trend is seen in *μ*, as shown in Table 3, where μ increases. The increase of *μ* and *D* is attributed to the improvement in chain flexibility with the presence of glycerol. When the amount of glycerol is increased, the values of *D*, *μ*, and *n* are increased, contributing to increasing conductivity. This is because the further glycerol insertion dissociates additional salts to free ions, thereby increasing the density of the ion carriers [48].

### 3.2. Dielectric Properties

The complex permittivity function (ε^*^(ω) = ε’(ω)+ε”(ω)) is the property of materials that depends on the frequency of the applied field, temperature, and structure and composition of polymer electrolytes [57,58,59]. It has been confirmed that the study of dielectric constant is the crucial parameter for determining the conductivity behavior of polymer electrolytes and understanding the mechanism of ion transport [34,60,61,62]. The dielectric constant (ɛ’) and dielectric loss (ɛ”) were determined using Equations (2) and (3).

**Figure 2:**
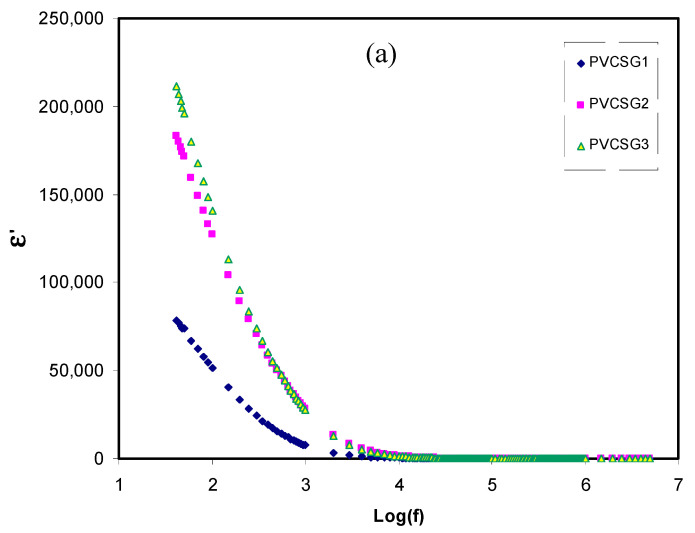

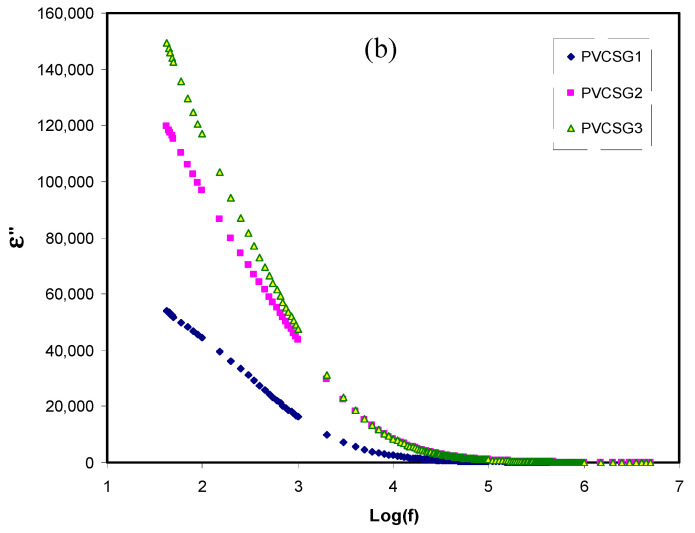
Dielectric parameters of (**a**) dielectric constant and (**b**) dielectric loss vs. log(f) for all plasticized electrolyte samples.

Figure 2a,b exhibit the continuous variation of dielectric constant (ɛ’) and dielectric loss (ɛ”) over the frequency range of each plasticized film. It is seen that the value of ɛ’ reaches its maximum value in the low-frequency region. Such maximum value of ɛ’ at the low-frequency can be correlated to the charge accumulation at the interfacial region [32,33,62]. It is also observed that the value of ɛ’ begin to drop in the high-frequency region. This dropping in ɛ’ value is attributed to the limitation of ion diffusion. It is also significant that the dipole molecules do not have enough time to change their orientation in the applied electrical field [60,61,62]. It is seen that the ɛ’ reaches the maximum value at 42 wt % of glycerol, and then reduces the value for other amounts of glycerol. Profoundly, the high ɛ’ is a decisive factor where the salt’s dissociation means more ions participating in polarization and the conduction process [34,62].

There is a function between DC conductivity and ɛ’ in one side and ion movement and polarization on the other side. It is seen that the trends of ɛ’ and DC conductivity are similar, indicating that ɛ’ investigation is a proper property from which one can tackle the conductivity behavior of polymer electrolytes. It is well-established, mathematically, that the carrier density is directly connected to the dielectric constant (ɛ′) and dissociation energy (*U*), which can be understood through the relationship

(12) $$n=\;n_{o}\;\exp\left(-\frac{U}{\varepsilon^{\prime }KT}\right)$$

The increase in the ɛ′ value with glycerol addition results in an increase in charge carrier concentration and DC conductivity [4,32,34].

### 3.3. TNM and LSV Studies

**Figure 3:**
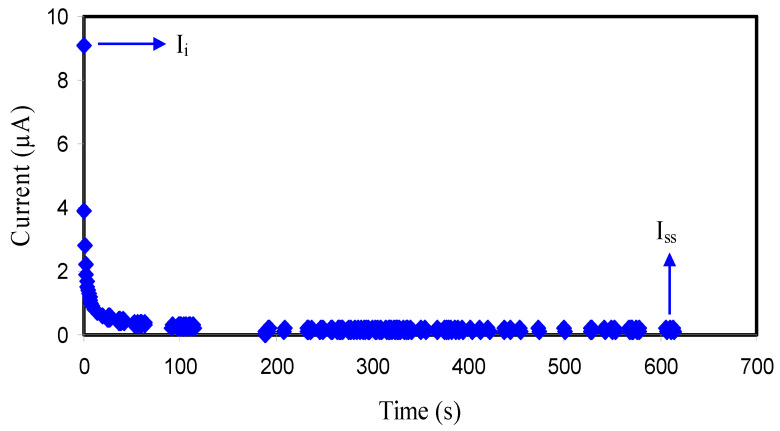
Variation of current with respect to time for the cell assembled with SS/CS:PVA:LiClO_4_:glycerol/SS.

Utilization of benign electrolytes, particularly solid polymer electrolytes (SPEs) is of great importance in electrochemical device application [63,64]. Although SPEs were introduced and studied since the late 1970s, they have still been considered as one of the leading topics [1,47]. It is necessary to perform the transference number analysis (TNM) and linear sweep voltammetry (LSV) to test polymer electrolytes for use in large scale applications. From the TNM analysis, one can determine the dominant charge carrier within polymer electrolytes. The ion transference number (t_ion_) of polymer electrolyte samples can be examined via DC polarization technique. In this technique, DC voltages are scanned in an electrochemical cell sample within the potential window, followed by recording corresponding current values in an experimental time scale (see Figure 3) [65]. In the electrochemical cell, the relatively high conductive polymer electrolyte was placed between a pair of stainless steel blocking electrodes (SS) to perform electrochemical measurements including both the ion (t_ion_) and the electron (t_e_) transference numbers using the relationship [31,48,55]

(13) $$t_{ion}=\frac{I_{i}-I_{ss}}{I_{i}}$$

(14) $$t_{e}=1-t_{ion}$$

where *I_ss_* and *I_i_* are the steady-state current and initial current, respectively. The immense value of the initial current can be attributed to the contribution of both ion and electron [53,66,67]. It is known that the used electrodes are stainless steels which prevent ion-transport (ion blocking phenomenon); thereby, a substantial drop of current is noticed before steady-state constant current value at 0.1 μA. This phenomenon indicates the behavior of an ionic nature of the polymer electrolyte system [68]. Moreover, the values of *t_ion_* = 0.989 and *t_el_* = 0.011 confirm dominancy of ion to carry charge within the polymer electrolyte. Furthermore, the proximity of *t_ion_* to 1 as ideal value is actually crucial for dealing with the mechanism of conduction in such particular polymer electrolyte system [65,69,70].

**Figure 4:**
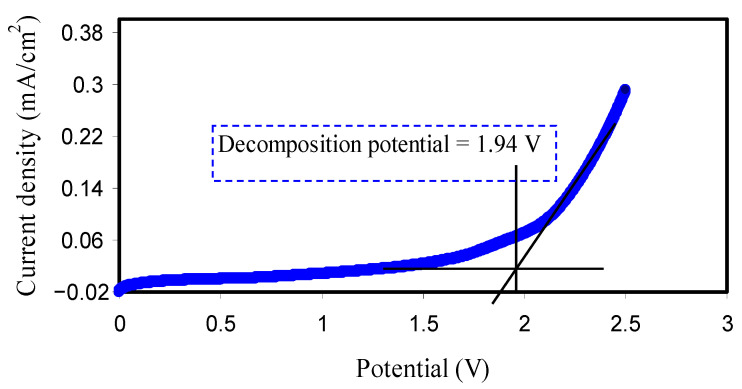
LSV curve for the cell with SS/CS:PVA:LiClO_4_:glycerol/SS at scan rate of 10 mV/s at ambient temperature.

The voltage window of the polymer electrolyte system is of vital importance in terms of large-scale utilization [71,72]. The LSV was applied as an efficient technique to determine the voltage window polymer electrolyte. Figure 4 presents a typical LSV response of relatively high conducting CS:PVA:LiClO_4_ blend electrolyte. It is seen that the voltage window range of the polymer blend electrolyte is 1.94 V that is satisfactory to be utilized in EDLC [73]. Monisha et al. [74] have noted that the threshold voltage is the flow of current through the cells. Bockenfeld et al. [75] used protic ionic liquids as electrolyte for lithium-ion batteries. The authors indicated that the electrolyte 0.5 M lithium nitrate (LiNO_3_) in propylene carbonate (PC)-pyrrolidinium nitrate (PYRNO_3_) displayed the overall electrochemical stability window of 2.65 V. They illustrated that the electrochemical stability value is large enough to guarantee a safe extraction and insertion of lithium into lithium iron phosphate (LiFePO_4_, LFP) electrode.

From the DC conductivity and transference number of ions (t_ion_) values, the diffusion coefficient and mobility of cations and anions of all polymer electrolytes were calculated by the following equations [76].

(15) $$D=kT\sigma /ne^{2}$$

(16) $$D=\;D_{+}+\;D_{-}$$

(17) $$t_{ion}=\;D_{+}/\left(D_{+}+\;D_{-}\right)$$

(18) μ= σ/ne

(19) $$\mu =\;\mu_{+}+\;\mu_{-}$$

(20) $$t_{ion}=\;\mu_{+}/\left(\mu_{+}+\;\mu_{-}\right)$$

where *k* is Boltzmann constant, *T* is the absolute temperature, *σ* is the conductivity, and *e* is the charge of electrons, *D*_+_ is the diffusion coefficient of cation, *D*_−_ is the diffusion coefficient of anion, *μ*_+_ is the mobility of cation and *μ*_−_ is the mobility of anion. The size of cations are smaller than the anions and this is a reason why *μ*_+_ is larger than the *μ*_−_. Table 3 shows that the *D*_+_ and *μ*_+_ are greater than the *D*_−_ and *μ*_−_, respectively. When the conductivity increases, the *μ*_+_ and *μ*_−_ also increase and vice versa. The similar behavior is reflected for *D*_+_ and *D*_−_. Therefore, the transference number measurement (TNM) leads to the conclusion that the DC conductivity was influenced by *D*_+_ and *μ*_+_. The highest conducting plasticized electrolyte has the maximum value of ionic mobility and diffusion coefficient in comparison with the other plasticized electrolytes.

### 3.4. Cyclic Voltammetry Test for the EDLC Device

Cyclic voltammetry (CV) as an informative technique can be used to evaluate EDLCs in terms of qualitative and quantitative aspects [26,77]. Minakshi et al. [78] illustrated that the electrochemical energy storage of sodium and lithium ions from aqueous solution in binary metal oxides is vital for applications in renewable energy storage. They described the binary metal oxides as novel electrode materials for supercapacitors. The authors used CV to evaluate the electrochemical behavior of the symmetric capacitor. Biswal et al. [79] synthesized hierarchical porous cobalt–nickel–iron (Co–Ni–Fe) ternary oxide heterostructure as an electrode for hybrid capacitor application owing to their high redox potentials. They investigated the electrochemical behavior of the Co–Ni–Fe ternary hydroxides using CV at different scan rates. The CV curve showed an elliptical at high scan rates, deviated from rectangular shape, representing a large resistance of the fabricated material has a dominant role and a slight contribution from electron transfer reaction of electrolyte ions at the ternary oxides interface.

**Figure 5:**
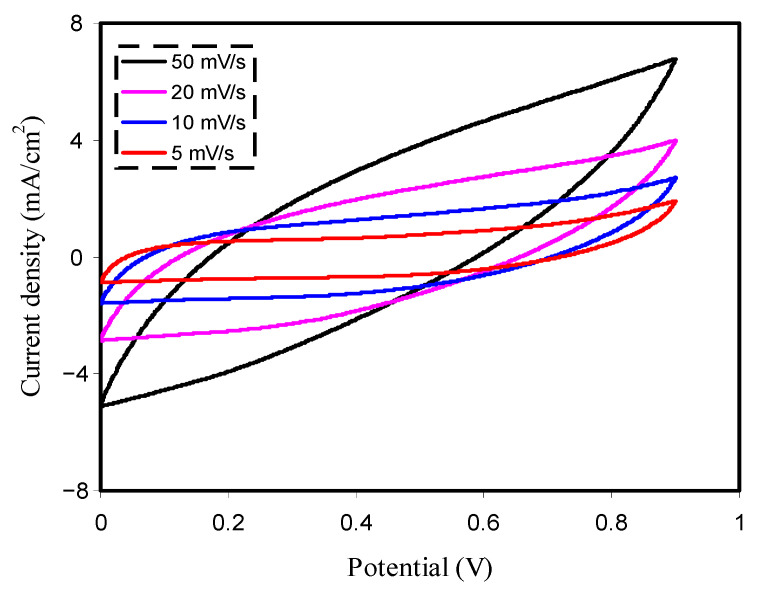
Cyclic voltammetry (CV) plot of the fabricated EDLC for PVCSG3 film in the potential range of 0–0.9 V.

In our study, the CV was carried out at sweep rate of 5, 10, 20, and 50 mV/s as shown in Figure 5. It is seen from the CV response that there is no redox peak (Faradaic process) in the voltage range of 0 to 0.9 V. Instead, the non-Faradaic response occurs as a result of electrical double layer formation [16,80]. This phenomenon is desired in EDLCs and supercapacitors [81]. This also rationalizes the charging storage process mechanism in EDLC as a consequence of ion accumulation at the electrode–electrolyte interface in response to an electric potential application [51,82]. There is a deviation of the rectangular shape of the CV response at higher scan rates, as shown in Figure 5. According to Panday and coworkers, the deviation from the perfect rectangular shape results from the gradual build-up of the electric double layer [83]. The CV curve deviates from rectangular to elliptical shape, which is due to carbon porosity and internal resistance, thus resulting to a current–voltage dependence [48]. Brza et al. [48] showed that the ions migrate very fast toward the surface of the electrodes at higher scan rates and thus the ions cannot form a proper double layer. This is why the current is not stable and it depends on the voltage. The specific capacitance (*C_spe_*) of the EDLC assembly can be obtained from the CV response via the equation [16,31]

(21) $$C_{spe}=\int_{V_{i}}^{V_{f}}\frac{I\left(V\right)dV}{2mv\left(V_{f}-V_{i}\right)}$$

where *I(V)dV* refers to the area of the CV response, which is calculated by Origin 9.0 software via the integration function. In this work, *V_i_* and *V_f_* are set to 0 and 0.9 V, respectively. The *m* and *v* parameters refer to the mass of active material used and sweep rate, respectively. Table 4 presents the values of *C_spe_* obtained from the CV. The values can be compared to those derived from the charge–discharge graph of the EDLC in the next section.

### 3.5. Galvanostatic Charge–Discharge (CDG) Study

**Figure 6:**
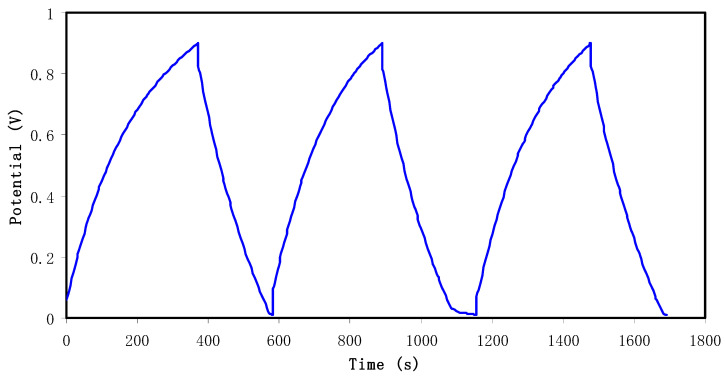
Charge–discharge profiles of the fabricated EDLC at selected cycles.

**Figure 7:**
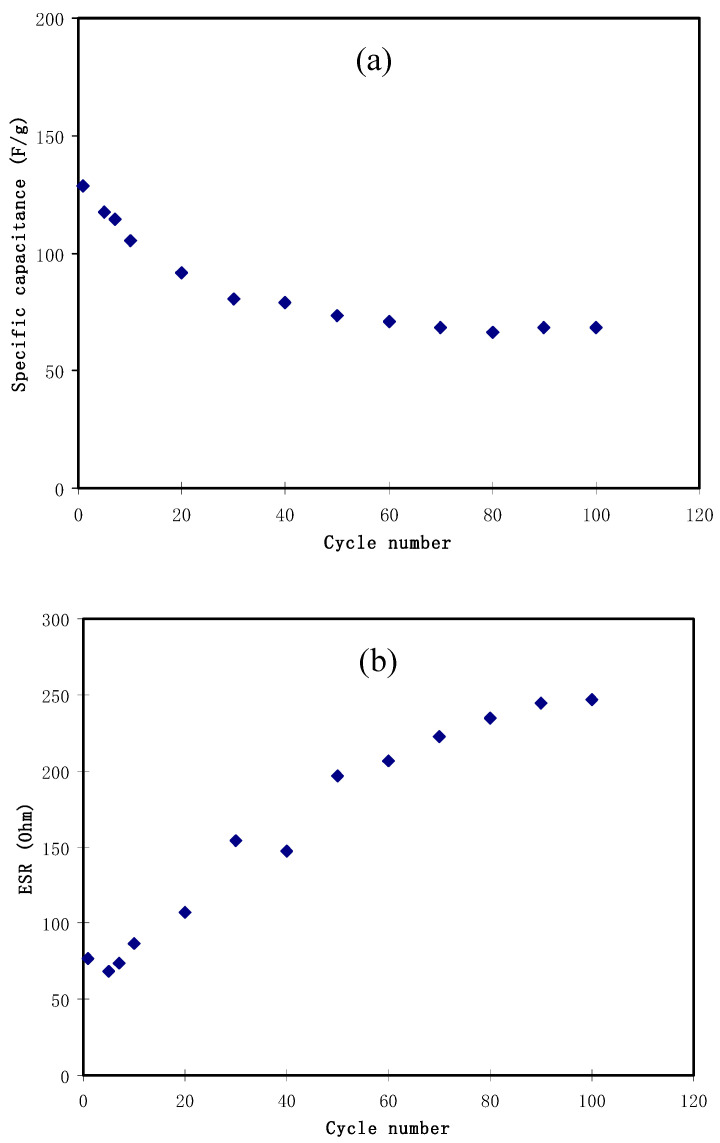
EDLC parameters of (**a**) specific capacitance and (**b**) ESR at 0.5 mA/cm^2^ for 100 complete cycles.

The charge–discharge profiles of the fabricated EDLC were obtained by holding the current density at 0.5 mA cm^−2^ in the potential range of 0 to 0.9 V, as shown in Figure 6. The discharge/charge current used to obtain the CD curves is 1 mA. The slope of the discharge response is nearly linear, confirming the capacitive behavior of the EDLC [84,85]. The specific capacitance (*C_s_*) can be determined from this slope using the equation [31]

(22) $$C_{S}=\frac{i}{sm}$$

where *i* is the constant current, s is the slope of the GCD discharge line and *m* is the mass of active material (active carbon). The *C_s_* values of the EDLC within 100 cycles have been determined and shown in Figure 7a. At the first cycle, the *C_s_* value is 130 F g^−1^. It drops to 70 F g^−1^ at the 50th cycle and becomes constant with a value within 55 to 60 F g^−1^. This value is comparable to the *C_spe_* value obtained from the CV analysis at the scanning rate of 20 mV s^−1^ (Table 4).

The trend of decreasing of the C_s_ values at higher cycles indicates the depletion of the electrolyte where ion pairs are probably created and contribute to the electrochemical device’s instability. The ion pairs and ion aggregation formation cause the decrement in mobile charge carriers’ availability in the migration between the electrodes. Therefore, this will decrease the ion adsorption onto the electrodes [86].

A recent publication has shown that for unplasticized CS:PEO:LiClO_4_ polymer blend electrolyte has a specific capacitance of about 5 Fg^−1^ [87]. Teoh et al. [88] used free LiClO_4_ plasticizer based on corn starch polymer electrolyte in an EDLC assembly, and the value of *C_s_* was 7.1 F g^−1^. In another study, an EDLC assembly possesses specific capacitance values (2.6–3.0 and 1.7–2.1 F g^−1^) correspond to the Mg-based PEO and Li-based PEO polymer electrolytes incorporated with ionic liquids, respectively [77]. Importantly, the present capacitor has shown a capacitance greater than that reported (61.7 F g^−1^) for ionic liquid-based gel polymer electrolyte system carried out by Mukta Tripathi & S.K. Tripathi [65]. Moreover, the present specific capacitance value is comparable to that of the gel-based polymer electrolytes, 87.3 F g^−1^ and 90 F g^−1^ reported by Boonen et al. [89] and Łatoszyńska et al., respectively [90]. Bockenfeld et al. [75] used protic ionic liquids as electrolyte and lithium iron phosphate (LiFePO_4_, LFP) as electrode for lithium-ion batteries. The author indicated that the electrolyte 0.5 M lithium nitrate (LiNO_3_) in propylene carbonate (PC)-pyrrolidinium nitrate (PYRNO_3_) guarantees a good cycling stability. The LFP electrode was able to provide a capacity of 134 mAhg^−1^. Based on their results, they illustrated that the protonic ionic liquids as electrolytes for lithium-ion battery is possible.

The redox peaks show intercalation/ deintercalation or Faradaic process, which is different from the energy storage mechanism in EDLC. Li^+^ cation and ClO_4_^−^ anion migrate toward the electrodes in opposite directions to create the double layer capacitor. The EDLC accumulates energy within adsorption/desorption process or non-Faradaic processes [91,92,93]. Activated carbon as electrodes of EDLC have large surface area of 2500 m^2^/g. Large surface area of activated carbon permits more ions to be adsorbed and thus the double layer is created owing to the ions accumulation [48]. Minakshi et al. [94] investigated amorphous iron phosphate (FePO_4_) as a cathode material electrode for applications in battery using aqueous (potassium hydroxide or lithium hydroxide) electrolytes. The authors investigated lithium intercalation into an FePO_4_ cathode in an aqueous electrolyte and they identified the lithium intercalation mechanism for LiOH. Minakshi et al. [95] in another study described Sn-LiCoPO_4_ battery using aqueous lithium hydroxide electrolyte. They mainly investigated the LiCoPO_4_ cathode with Sn as an anode in aqueous LiOH electrolyte. The authors investigated Sn as the anode for aqueous secondary batteries so as to enhance the reversible capacity and cyclic efficiency in comparison with the Zn-based anode.

Another decisive parameter in EDLC is equivalent series resistance (*R_esr_*), which mathematically can be expressed in the relationship [48].

(23) $$R_{s}=\;\frac{V_{d}}{i}$$

Figure 7b presents the *R_esr_* of the EDLC within 100 cycles in which ranged from 80 to 250 Ω. There are three causes of internal resistance, which are resistance in the electrolyte, current collectors and current collector-electrolyte gap [70,96]. Importantly, low *R_esr_* value indicates sufficient contact between electrode and electrolyte, confirming ion migration facilitating bulk electrolyte to the electrode surface, resulting in an electrical double-layer formation [97]. The *R_esr_* of the EDLC in this study is relatively low compared to ionic liquid-based-PEO-based polymer electrolyte (i.e., 1300 Ω) [77].

Finally, other crucial parameters for the EDLC are energy (*E_den_*) and power (*P_den_*) densities. Energy density measures the quantity of energy that an EDLC can store. Power density is a measure of power that an EDLC can deliver [48,98]. Both parameters of *E_den_* and *P_den_* can be calculated from the relationships [31,48]

(24) $$E_{den}=\frac{C_{s}V}{\begin{array}{c} 2 \end{array}}$$

(25) $$P_{des}=\frac{V^{2}}{4m\left(ESR\right)}$$

**Figure 8:**
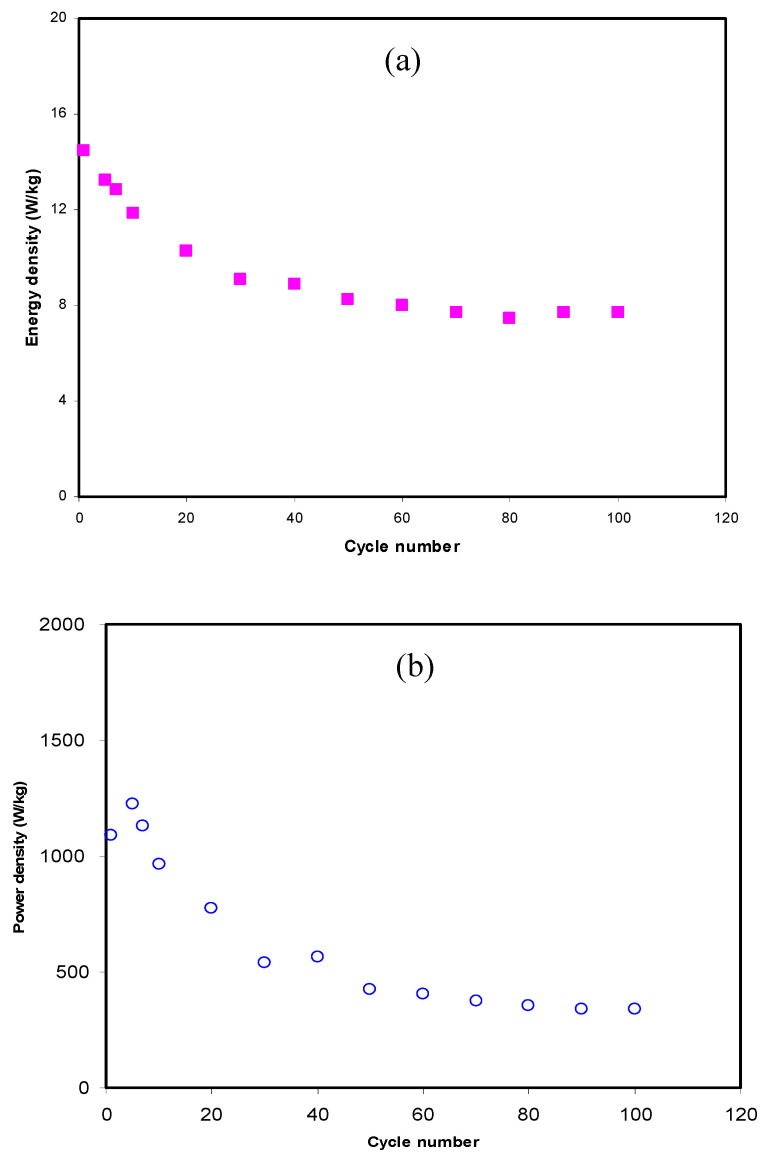
EDLC parameters of (**a**) energy density and (**b**) power density at 0.5 mA/cm^2^ throughout the 100 cycles.

In the present study, the applied voltage (V) on the cell assembled system of the EDLC was 0.9 V. Figure 8a,b show the estimated energy and power densities of the EDLC throughout the 100 cycles. The values of *E_den_* and *P_den_* at the 1st cycle are 14.5 Wh kg^−1^ and 1100 W kg^−1^, respectively. The two parameters drop to 8.8 Wh kg^−1^ and 500 W kg^−1^ at the 30th cycle. It is also noticed that beyond the 30th cycle to the 100th cycle, the energy and power densities become almost constant at 8.1 Wh kg^−1^ and 444 W kg^−1^, respectively. In an earlier study [87], the EDLC containing CS:dextran:NH_4_F as an electrolyte that separates the electrodes has shown 1.4 Wh kg^−1^ and 428 W kg^−1^ values, respectively. It is well-defined that in the amorphous region, the conduction of ions is dominant [99]. The uniform values of energy density indicate that the ions in the plasticized electrolyte system face the same energy barrier during the conduction process of the interface [100]. Generally, the performance lowering of the EDLC—i.e., *C_s_*, *E_den,_* and *P_den_* values at higher cycles—is mainly due to electrolyte depletion. The depletion phenomenon occurs during the rapid charge–discharge process, where ion recombination results in aggregation. This also causes the potential energy developed at the surface of the carbon electrodes to be decreased [80].

## 4. Conclusions

Preparation of lithium-based biopolymer electrolytes was carried out successfully using solution cast methodology. It is concluded that glycerol, as a plasticizer, improves the conductivity of polymer electrolyte considerably. The equivalent electric circuit (EEC) modeling helps us to rationalize the electrical character of the films. The sample doped with 42 wt % of glycerol shows the highest dielectric constant with the maximum ionic conductivity of 8.457 × 10^−4^ S/cm. As the concentration of glycerol increased, the mobility (*μ*), diffusion coefficient (*D*), and number density (*n*) of ions gradually increased. A strong correlation between dielectric parameter and DC conductivity has been established and verified to comprehend the charge transport mechanism within the polymer electrolyte system. The TNM analysis confirmed that the primary charge carriers were ions. t_ion_ and t_e_ values were identified to be 0.989 and 0.011, respectively, for the highest conducting plasticized film. The LSV analysis showed that the decomposition voltage of the PVCSG3 system is 1.94 V, indicating the electrolyte suitability for the EDLC application. Ultimately, the blending methodology has provided the desired polymer electrolyte with high ionic conductivity and good electrochemical properties. At the first cycle, the internal resistance, specific capacitance, energy density, and power density were determined to be 80 Ω, 130 F/g, 14.5 Wh/kg, and 1100 W/kg, respectively.

## Figures and Tables

**Table 1:** EEC fitting parameters for PVA:CS: LiClO_4_: glycerol systems at room temperature.

Sample	P(rad)	K (F^−1^)	C (F)
PVCSG1	0.693	4.39 × 10^4^	2.28 × 10^−5^
PVCSG2	0.697	1.99 × 10^4^	5.03 × 10^−5^
PVCSG3	0.3716	1.10× 10^4^	9.09 × 10^−5^

**Table 2:** DC conductivity values for PVA:CS: LiClO_4_: glycerol systems.

Sample Designation	DC Conductivity (S/cm)
PVCSG1	1.409 × 10^−4^
PVCSG2	4.228 × 10^−4^
PVCSG3	8.457 × 10^−4^

**Table 3:** Values of ω and ion transport parameters at room temperature.

Sample	ω (rad s^−1^)	*D* (cm^2^ s^−1^)	*µ* (cm^2^ V^−1^ s)	*n* (cm^−3^)	*D*_+_ (cm^2^ s^−1^)	*D*_−_ (cm^2^ s^−1^)	*µ*_+_ (cm^2^ V^−1^ s)	*µ*_−_ (cm^2^ V^−1^ s)
PVCSG1	4.78 × 10^5^	4.59 × 10^−9^	1.79 × 10^−7^	4.92 × 10^21^	4.54 × 10^−9^	5.04 × 10^−11^	1.77 × 10^−7^	1.96 × 10^−9^
PVCSG2	5.65 × 10^5^	1.28 × 10^−8^	5.01 × 10^−7^	5.27 × 10^21^	1.27 × 10^−8^	1.41 × 10^−10^	4.95 × 10^−7^	5.51 × 10^−9^
PVCSG3	5.59 × 10^5^	2.12 × 10^−8^	8.25 ×10^−7^	6.4 × 10^21^	2.09 × 10^−8^	2.33 × 10^−10^	8.16 × 10^−7^	9.07 × 10^−9^

**Table 4:** C_spe_ value of the PVCSG3 film at the various scan rates of 50, 20, 10, and 5 mV/s.

Scan Rate (mV/s)	Capacitance (F/g)
50	31.09
20	57.61
10	80.70
5	94.65
